# HIF2α/EFEMP1 cascade mediates hypoxic effects on breast cancer stem cell hierarchy

**DOI:** 10.18632/oncotarget.9846

**Published:** 2016-06-06

**Authors:** Ji-Hye Kwak, Na-Hee Lee, Hwa-Yong Lee, In-Sun Hong, Jeong-Seok Nam

**Affiliations:** ^1^ Laboratory of Stem Cell Research, Lee Gil Ya Cancer and Diabetes Institute, Gachon University, Incheon, 406-840, Republic of Korea; ^2^ Department of Molecular Medicine, School of Medicine, Gachon University, Incheon, 406-840, Republic of Korea; ^3^ The Faculty of Liberal Arts, Jungwon University, Chungbuk, 367-805, Republic of Korea; ^4^ School of Life Sciences, Gwangju Institute of Science and Technology, Gwangju, 500-712, Republic of Korea

**Keywords:** breast cancer cancer stem cells, hypoxia, HIF2α, EFEMP1

## Abstract

Breast cancer stem cells (BCSCs) have been shown to contribute to tumor growth, metastasis, and recurrence. They are also markedly resistant to conventional cancer treatments, such as chemotherapy and radiation. Recent studies have suggested that hypoxia is one of the prominent micro-environmental factors that increase the self-renewal ability of BCSCs, partially by enhancing CSC phenotypes. Thus, the identification and development of new therapeutic approaches based on targeting the hypoxia-dependent responses in BCSCs is urgent. Through various *in vitro* studies, we found that hypoxia specifically up-regulates BCSC sphere formation and a subset of CD44^+^/CD24^−/low^ CSCs. Hypoxia inducible factors 2α (HIF2α) depletion suppressed CSC-like phenotypes and CSC-mediated drug resistance in breast cancer. Furthermore, the stimulatory effects of hypoxia-induced HIF2α on BCSC sphere formation were successfully attenuated by epidermal growth factor-containing fibulin-like extracellular matrix protein 1 (EFEMP1) knockdown. Taken together, these data suggest that HIF2α mediates hypoxia-induced cancer growth/metastasis and that EFEMP1 is a downstream effector of hypoxia-induced HIF2α during breast tumorigenesis.

## INTRODUCTION

Local oxygen (O_2_) concentrations can directly influence the differentiation or self-renewal capacity of stem cells. Recent *in vitro* evidence indicates that hypoxia, defined as reduced oxygen tension, promotes an undifferentiated and multipotent status in human embryonic [1] and adult [2] stem cells. Although it is certainly a valid generalization that severe or prolonged hypoxia is generally toxic for both normal and tumor tissues, cancer cells gradually adapt to chronic hypoxia though positive or negative regulation of hypoxia-inducible factors with a net result that hypoxia strongly promotes poor patient survival, therapeutic resistance and an aggressive tumor phenotype [3]. Recently, it was suggested that a subset of tumor cells known as cancer stem cells (CSCs) contribute to tumor growth, metastasis, and recurrence [4]. Importantly, CSCs have been shown to be resistant to conventional therapies, such as chemotherapy [5] and radiation [6]. Furthermore, it has been reported that hypoxia increases the CSC subpopulations and promotes the acquisition of a CSC- like phenotype [7], thereby aggravating the patient's prognosis. Therefore, these stimulatory effects of hypoxia on tumorigenesis prompted us to investigate the potential mechanisms by which hypoxia stimulate the tumorigenic properties of CSCs.

Cancer cells have regulatory mechanisms to quickly respond to changes in oxygen tension within cells/tissues using the transcription factor known as hypoxia inducible factors (HIFs). The HIFs, which are heterodimer molecules consisting of an alpha subunit and a beta subunit, have been recognized as the master regulators of hypoxia-induced changes [8]. Though HIF1α and HIF2α share a high degree of sequence homology, most studies investigating the mechanisms of hypoxia-induced effects have been focused on HIF1α largely due to its earlier discovery and more ubiquitous expression pattern in tissues compared with HIF2α, which demonstrates more restricted expression [9]. However, recent experimental evidence has demonstrated that HIF2α is only significantly present in the CSC subpopulation [10] and promotes tumor proliferation and radiation resistance [11, 12]. Furthermore, Pahlman and his colleagues demonstrated that the high expression of HIF2α correlates with immature phenotypic features and poor outcome in patients undergoing brain tumor surgery [13]. Moreover, hypoxia-induced HIF2α can increase the expression of stem cell-related markers and confer tumorigenic potential to non-CSCs of human brain cancers [14]. Intriguingly, recent advances in cancer research have revealed that hypoxia-induced HIF2α, but not HIF1α, promotes hypoxic cell proliferation by enhancing the expression of Oct4 [15] and the transcriptional activity of c-Myc [11]. Because both Oct4 and c-Myc are well-known factors for maintaining and re-establishing pluripotency, these data shed light on how hypoxia-induced HIF2α stimulates the tumorigenic potential of CSCs. Nonetheless, the role of hypoxia-induced HIF2α in CSC tumorigenesis and the potential mechanism by which HIF2α is increased during tumorigenesis under hypoxic conditions remain unclear.

EFEMP1 (epidermal growth factor-containing fibulin-like extracellular matrix protein 1), which is also known as Fibulin-3, is a member of the fibulin family of extracellular matrix (ECM) glycoproteins [16]. The fibulin family is widely distributed and is often associated with vasculature and elastic tissues, whose major function is to mediate homotypic interactions among cells and heterotopic cell-matrix interactions [17]. It has been previously reported that fibulin family members 1, 2, 4, and 5 play crucial roles in the promotion of tumorigenesis [16]. However, the relationship between EFEMP1 and HIF2α during hypoxia-induced tumorigenesis remains unclear. Because HIF signaling mediates increased fibulin expression under hypoxic conditions [18] and because fibulins seem to play an instrumental role in breast cancer chemoresistance [19], we further investigated whether hypoxia-induced EFEMP1 influences the tumorigenic properties of CSCs as a both key regulator of hypoxia and a downstream effector of hypoxia-induced HIF2α.

Here, we showed that hypoxia specifically up-regulates a subset of *CD44*^+^/*CD24*^−/low^ *CSCs*. Through various *in vitro* studies, we found that HIF2α depletion suppressed CSC-like phenotypes and CSC-mediated drug resistance in breast cancer. Moreover, dysregulation of EFEMP1 expression has been reported to correlate with poor prognosis and lymph node metastasis in cervical cancer patients [20]. Although EFEMP1 seems to be important in the development of breast cancer, its role in BCSC functions remains unknown. We therefore demonstrated, for the first time, that hypoxia-induced BCSC sphere formation (*CD44*^+^/*CD24*^−/low^ *subpopulation) in vitro* and tumor formation *in vivo were markedly suppressed* by EFEMP1 knockdown. Taken together, these data suggest that HIF2α, but not HIF1α, mediates hypoxia-induced breast cancer growth and that EFEMP1 promotes BCSC renewal and tumor metastasis as a downstream effector of hypoxia-induced HIF2α during breast tumorigenesis.

## RESULTS

### A subpopulation of stem-like cancer cells is enriched by hypoxic conditions

**Figure 1:**
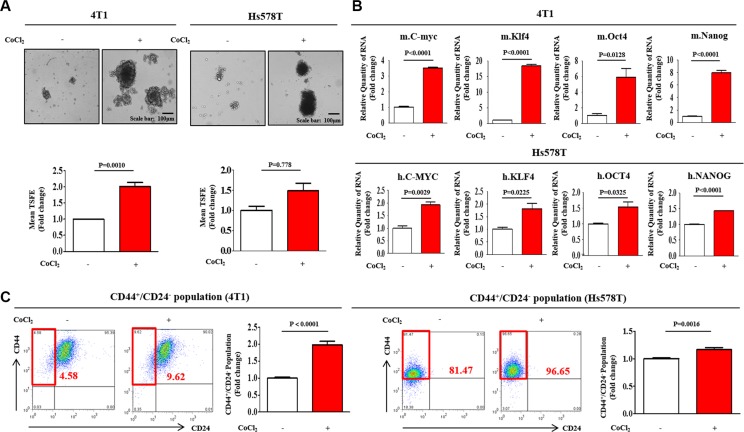
Effect of hypoxia on the growth and stemness-related features of BCSCs (**A**) Cobalt chloride (CoCl_2_) daily treatment stimulated BCSC sphere formation in both mouse (4T1) and human (Hs578T) breast cancer cell lines after one-week sphere culture. The sizes of spheres greater than 100 μm were enumerated, with a representative image of a tumorsphere shown. The data represent an average of three independent experiments. (**B**) Real-time PCR results demonstrating changes in the expression of the stem cell markers c-Myc, Klf4, Oct4, and Nanog by hypoxia induced with 100 μM CoCl_2_ for 24 h in both 4T1 and Hs578T cells. (**C**) The results of FACS analysis showing the percentage of the total cell population that consisted of CD44^+^/CD24^−^ cells in both 4T1 and Hs578T cells. The results represent the means ± SD from three independent experiments.

It has been suggested that three-dimensional (3D) sphere cultures demonstrated an enrichment of the cancer stem/progenitor cell population in different types of cancers, including breast [21], colon [22], and pancreatic cancers [23]. Recent studies have suggested that the stem cell markers c-Myc [24], Klf4 [25], Oct4 [26], and Nanog [27] play important roles in maintaining BCSC pluripotency. Here, we established a sphere-forming culture system for use as an *in vitro* model for BCSC culture using our published protocols [28]. To confirm whether sphere-forming subpopulations and stem cell-like properties were enriched under hypoxic conditions, we evaluated the effects of hypoxia on sphere formation and the expression profiles of the stem cell markers c-Myc, Klf4, Oct4, and Nanog. The number and size of the spherical colonies were significantly increased by hypoxia induced with cobalt chloride (CoCl_2_) in both mouse (4T1) and human (Hs578T) breast cancer cell lines (Figure 1A). Consistent with the sphere-formation results, the expression levels of stem cell markers were increased in CoCl_2_-treated cells compared with normoxic cells (Figure 1B). We also performed FACS analysis to quantitate the percentage of the total cell population with the CD44^+^/CD24^−^ phenotype during both hypoxia and normoxia. As expected, the relative percentage of cells expressing these surface markers was markedly increased in CoCl_2_-treated cells compared with normoxic cells (Figure 1C).

### Hypoxia stimulates the growth and stemness-related features of BCSCs through HIF2α activity

**Figure 2:**
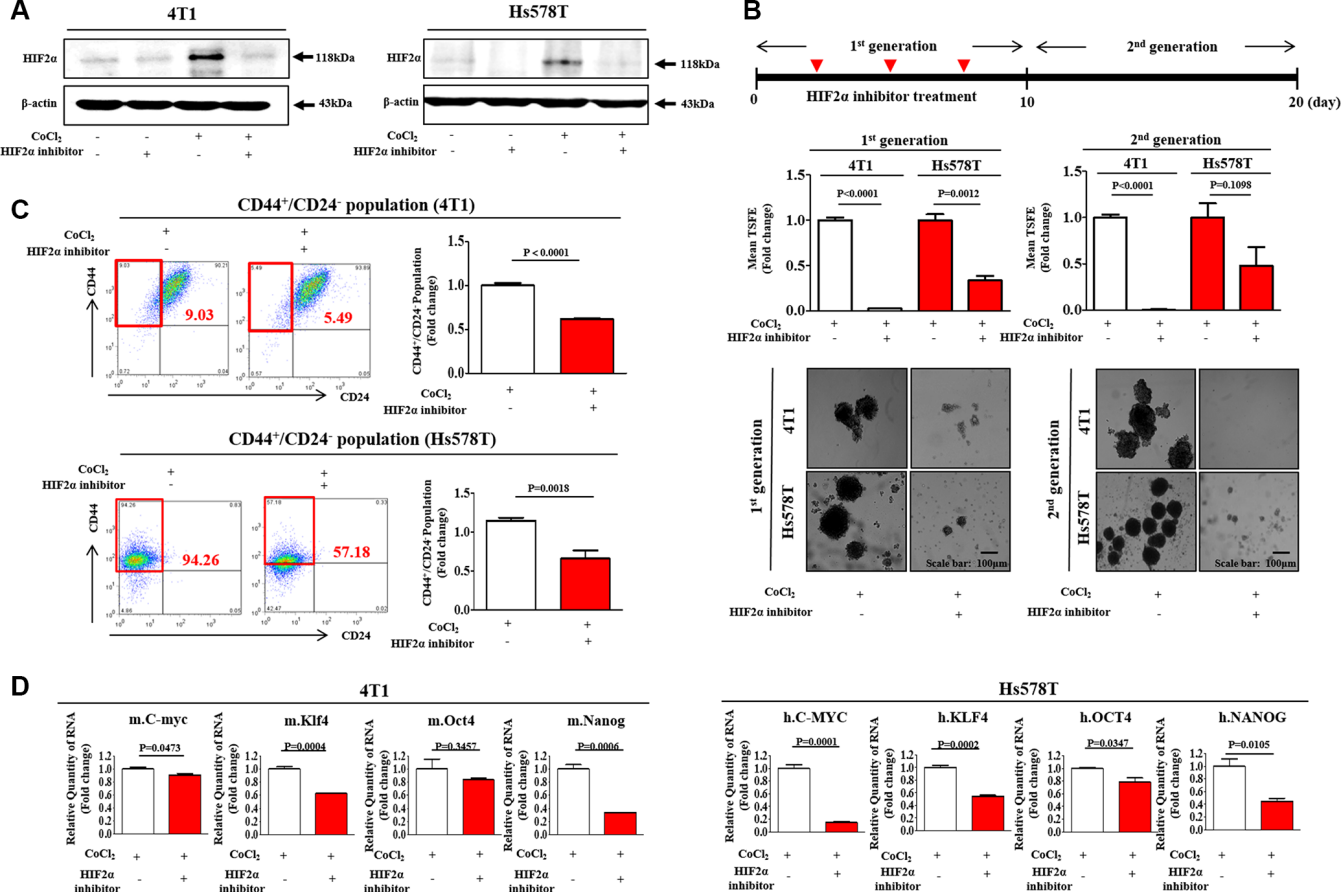
HIF2α inhibitor 76 suppressed CoCl_2_-induced immature phenotypic characteristics of BCSCs (**A**) The inhibitory effect of small molecule HIF2α inhibitor 76 for 24h (10 μM for 4T1 cells; 25 μM for Hs578T cells) on CoCl_2_-induced expression of HIF2α was assessed in both 4T1 and Hs578T cells by western blot analysis. (**B**) HIF2α inhibitor 76 inhibited primary (with HIF2α inhibitor 76 daily treatment) and second sphere formation (without additional HIF2α inhibitor 76 treatment) in both 4T1 and Hs578T cells. The sizes of spheres greater than 100 μm were enumerated, with a representative image of a tumor-sphere shown. The data represents an average of three independent experiments. (**C**) Treatment of 4T1 and Hs578T cells with HIF2α inhibitor 76 for 24 h led to a decrease in the percentage of CD44^+^/CD24^−^-positive cells as a proportion of total cancer cells. (**D**) 4T1 and Hs578T cells treated with CoCl_2_ (100 μM) for 24h and HIF2α inhibitor 76 for 24h (10 μM for 4T1 cells; 25 μM for Hs578T cells) either alone or together were evaluated for the expression levels of stem cell markers c-Myc, Klf4, Oct4, and Nanog by Real-time PCR. Abbreviations: TSFE, Tumor sphere-forming efficiency. β-actin was used as the internal control. The results represent the mean ± SD from three independent experiments.

Under hypoxic conditions, the aberrant expression or transcriptional activity of HIF2α has been associated with cancer cell amplification and tumorigenesis in a number of studies [11, 14, 15]. Consistent with these results, our previous study also demonstrated that HIF2α, but not HIF1α, mediates the hypoxia-induced stimulation of stemness-related features in BCSCs [28]. Therefore, it is quite reasonable to hypothesize that hypoxia stimulates the growth of BCSCs through the activation of HIF2α activity. To determine whether CoCl2 treatment mimics actual hypoxia-induced HIF2α expressions in our experimental conditions, we performed western blotting to evaluate the expression levels of HIF2α with or without CoCl2 treatment or hypoxic exposure. As expected, HIF2α expression is consistently increased by either CoCl2 treatment or hypoxic exposure in a hypoxic chamber. (Supplementary Figure 1). Using western blot analysis, we evaluated whether the small molecule HIF2α inhibitor 76 was sufficient to inhibit hypoxia-induced HIF2α expression in both 4T1 and Hs578T cells. Approximate IC_50_ values were determined using a dose-response curve. In both 4T1 and Hs578T cells, the IC_50_ values were 59 and 57 μM, respectively (Supplementary Figure 2). Pre-treatment of cells with the small molecule HIF2α inhibitor 76 potently decreased the CoCl_2_-induced up-regulation of HIF2α expression (Figure 2A). Additionally, to determine whether the blockade of HIF2α activity affects HIF1α expression in both 4T1 and Hs578T cells, we performed western blotting analysis to investigate the expression levels of HIF1α with or without HIF2α-specific inhibitor treatment. As expected, the stimulatory effects of CoCl_2_ on HIF1α expression were not affected by HIF2α-specific inhibitor treatment in both 4T1 and Hs578T cells (Supplementary Figure 3).

As a functional assay, we evaluated the effect of HIF2α inhibitor 76 on primary and secondary sphere formation. Treatment with HIF2α inhibitor 76 resulted in the disruption of CoCl_2_-induced primary sphere formation in both 4T1 and Hs578T cells (Figure 2B). For the secondary sphere-forming assay, treated primary spheres were collected and dissociated into single cells. The cells from treated or untreated primary spheres were re-plated on culture dishes without additional treatment. Interestingly, we observed that in the presence of the HIF2α inhibitor 76, the cells derived from primary spheres did not form secondary spheres as efficiently as the cells from untreated spheres did (Figure 2B). In breast carcinomas, the CD44^+^/CD24^−^ cell subset is thought to represent a stem cell-like population and is enriched in tumorigenic stem/progenitor cells [29]. Therefore, we hypothesized that HIF2α inhibitor 76 might disrupt hypoxia-induced BCSC sphere formation by targeting CD44^+^/CD24^−^ subpopulations. To test this hypothesis, we used FACS analysis to investigate the effect of HIF2α inhibitor 76 on CD44^+^/CD24^−^ subpopulations. Indeed, the treatment of 4T1 and Hs578T cells with HIF2α inhibitor 76 for 24 h decreased the size of CoCl_2_-induced CD44^+^/CD24^−^ subpopulation (Figure 2C). In breast carcinomas, the ALDH positive subset is thought to represent a stem cell-like population and is enriched in tumorigenic stem/progenitor cells. Consistently, the treatment of Hs578T cells with HIF2α inhibitor 76 for 24h decreased the size of the ALDH positive subpopulation (Supplementary Figure 4). Next, we examined the expression of BCSC markers in the presence or absence of HIF2α inhibitor 76. Consistent with our hypothesis, CoCl_2_-induced expression levels of c-Myc, Klf4, Oct4, and Nanog were significantly lower after HIF2α inhibitor 76 treatment than they were before (Figure 2D). Furthermore, we also investigated the stimulatory effects of CoCl_2_ exposure on the BCSC sphere formation (Supplementary Figure 5A), the size of the CD44+/CD24– subpopulation (Supplementary Figure 5B), and the expression of HIF1α and HIF2α (Supplementary Figure 5C) with or without HIF2α inhibitor 76 treatment in other liable human breast cancer cell lines such as MDA-MB-231.

### HIF2α depletion suppresses BCSC growth and chemo-resistance

**Figure 3:**
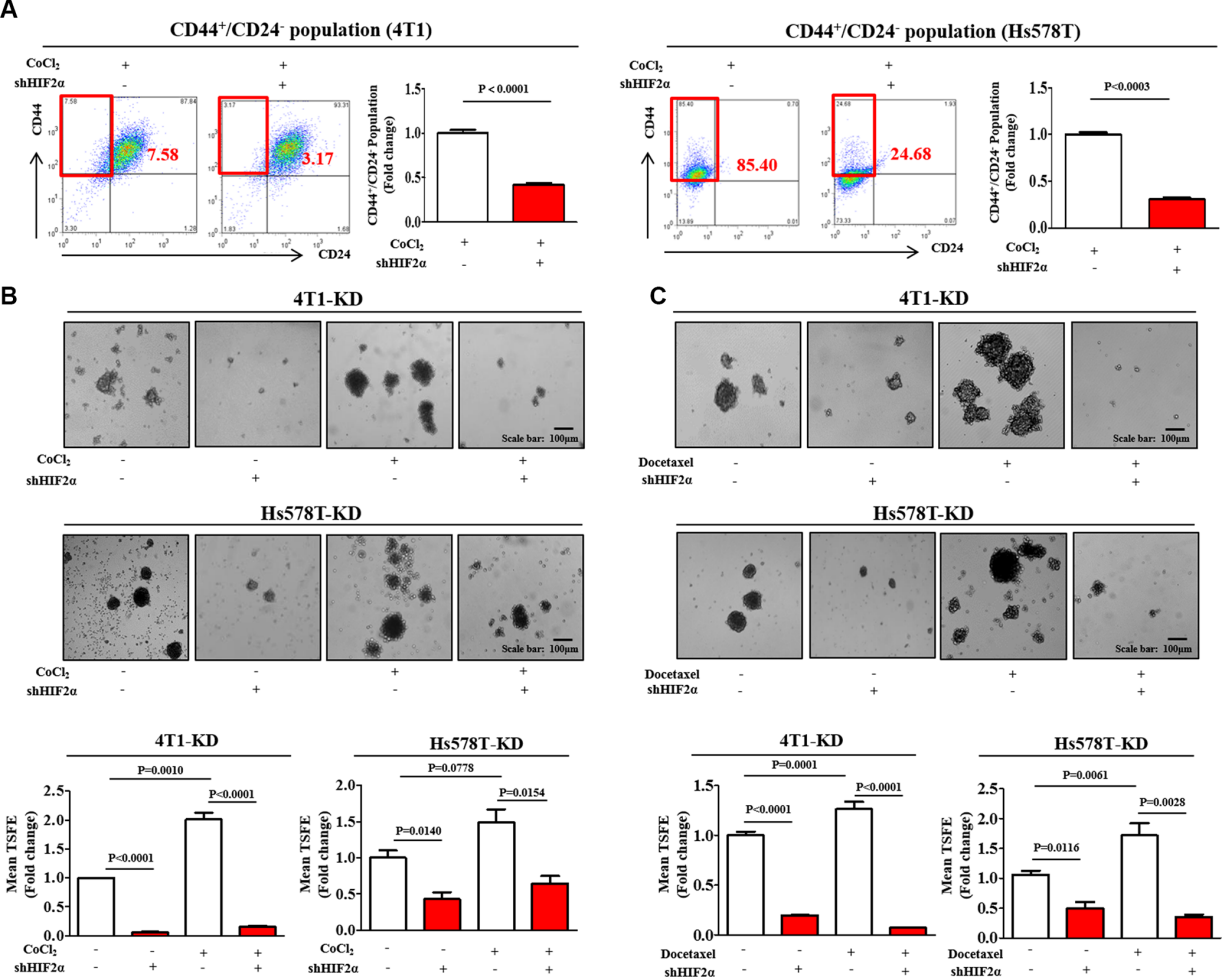
HIF2α knockdown suppresses BCSC growth and chemo-resistance (**A**) HIF2α knockdown using specific shRNA led to a decrease in the percentage of CD44^+^/CD24^−^ cells as a proportion of the total cancer cells under hypoxic conditions in both 4T1 and Hs578T cells. (**B**) The stimulatory effects of CoCl_2_ on BCSC sphere formation were successfully attenuated by HIF2α knockdown in both 4T1 and Hs578T cells. (**C**) Primary mammospheres from 4T1 and Hs578T cells treated with docetaxel (5 nM) for 24 h and HIF2α shRNA either alone or together were evaluated for the relative numbers of sphere-forming units. Abbreviations: TSFE, Tumor sphere-forming efficiency. The results represent mean ± SD from three independent experiments.

To further confirm the results observed using small molecule HIF2α inhibitor 76, we next knocked down HIF2α using specific shRNA in both 4T1 and Hs578T cells. Both 4T1 and Hs578T cells were transiently transduced with shRNA #1, #2, #3, #4, or #5 targeting HIF2α. HIF2α shRNA construct #2, hereafter referred to as HIF2α shRNA and, was the most efficient at knockdown. After then, stable cell lines with HIF2α knock-down was established using a lentivirus system. Successful knockdown of HIF2α were verified based on RNA and protein levels in both 4T1 and Hs578T cells (Supplementary Figure 6). Consistent with the results obtained from HIF2α inhibitor 76 treatment, HIF2α knockdown led to a significant decrease in CoCl_2_-induced CD44^+^/CD24^−^ subpopulations (Figure 3A) in both 4T1 and Hs578T cells. The stimulatory effects of CoCl_2_ on BCSC sphere formation were successfully attenuated by HIF2α knockdown in both 4T1 and Hs578T cells (Figure 3B). Interestingly, the stimulatory effects of hypoxia on HIF1α expression were slightly attenuated by HIF2α specific shRNA transection (Supplementary Figure 7). These results suggest that HIF2α may be at least partly involved in HIF1α-mediated hypoxic effects. In contrast with the results from HIF2α specific shRNA transection studies, these effects were not affected by HIF2α-specific inhibitor treatment in both 4T1 and Hs578T cells (Supplementary Figure 3). Therefore, further studies are warranted to investigate the functional Interaction between HIF1α and HIF2α under hypoxia in more detail.

Recently, it has been suggested that BCSCs are resistant to many conventional therapeutic approaches, including chemotherapy [30] and radiotherapy [31]. Thus, although traditional approaches may kill the majority of tumor cells, some BCSCs remain impervious to treatment and instead survive and generate new tumors. Consistent with previous studies [32], we found that both the relative size and the sphere forming abilities of 4T1 and Hs578T cells increased with conventional docetaxel treatment. However, these docetaxel-enriched sphere formations were markedly reduced by HIF2α knockdown (Figure 3C). Additionally, we conducted the additional three set of experiments to investigate the potential role of docetaxel treatment in the expressions of HIF1α and HIF2α in both 4T1 and Hs578T cells. Interestingly, docetaxel moderately increased HIF2α expression, but not HIF1α, in 4T1 cells However, HIF1α and HIF2α expressions were not affected by docetaxel in Hs578T cells (Supplementary Figure 8).

### Stimulatory effects of HIF2α on BCSCs are achieved through the upregulation of EFEMP1 activity

**Figure 4:**
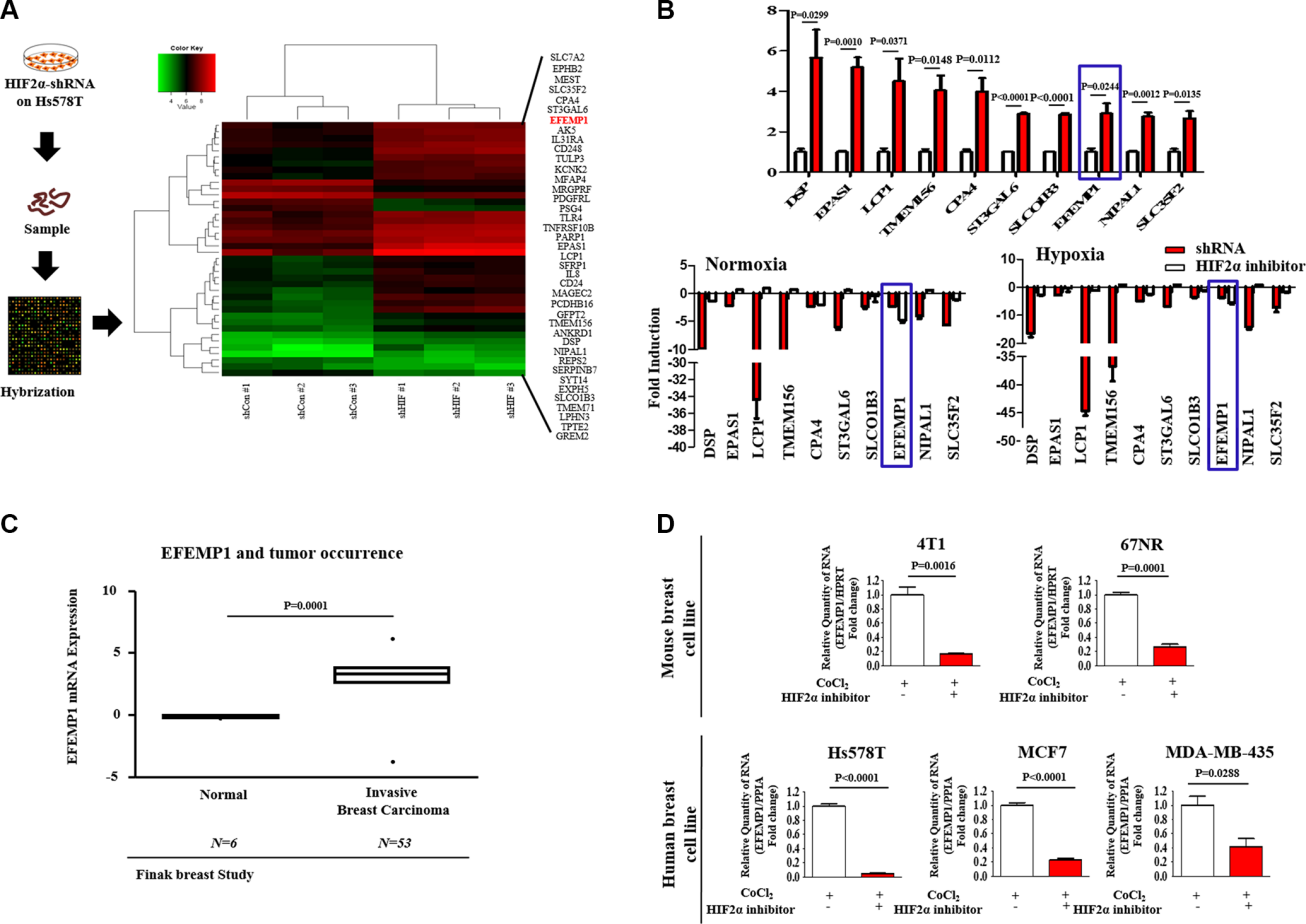
The expression levels of EFEMP1 are significantly suppressed by HIF2α knockdown (**A**) The data from genome-wide microarray analysis are presented as a density map of genes differentially expressed between wild-type and HIF2α knocked-down Hs578T cells with decreased (green) or increased (red) expression relative to the mean mRNA expression. The microarray array was analyzed using Ingenuity Pathway Analysis (IPA) software. (**B**) To verify the results obtained by microarray using quantitative real-time PCR (qPCR), Hs578T cells treated with or without HIF2α shRNA transfection for the expression level of EFEMP1. In quantitative real-time PCR (qPCR), which was used to verify the microarray results, HIF2α inhibitor 76 (25 μM) treatment for 24 h was correlated with decreased EFEMP1 levels (≥ 5.6- and 3.6-fold down-regulated, respectively) under hypoxic conditions. (**C**) A significant correlation between tumor reoccurrence and the expression of EFEMP1 was observed in human breast cancer datasets available through the Oncomine dataset repository (www.oncomine.org). (**D**) Multiple breast cancer cell lines (4T1, 67NR, Hs578T, MCF-7, and MDA-MB-435) treated with HIF2α inhibitor 76 (25 μM) for 24 h under CoCl_2_ treatment were evaluated for EFEMP1 expression. The results represent the mean ± SD from three independent experiments.

We compared differential gene expression in wild-type and HIF2α knockdown to identify potential downstream targets of hypoxia-induced HIF2α using a genome-wide microarray approach. We identified a subset of genes whose expression is affected by HIF2α-depletion, indicating that these genes may be targeted as downstream effectors of HIF2α (Supplementary Table 1). Two criteria for the selection of gene expression differences were employed: a significant *t*-test and fold-change magnitude. Among the genes examined, the level of EFEMP1 mRNA was significantly suppressed (≥ 3-fold change) in HIF2α knockdowns compared with shRNA controls (Figure 4A). Interestingly, in quantitative real-time PCR (qPCR), which was used to verify the microarray results, both HIF2α inhibitor 76 treatment and HIF2α knockdown were correlated with decreased EFEMP1 levels (≥ 5.6- and 3.6-fold down-regulated, respectively) under hypoxic conditions (Figure 4B). To investigate the association between hypoxia-induced tumorigenesis and EFEMP1 expression, we evaluated the available breast cancer datasets using the Oncomine dataset repository (www.oncomine.org). After specifically filtering for breast cancer datasets showing a frequency of tumor reoccurrence, we observed significant correlations between EFEMP1 upregulation and a higher incidence of breast cancer reoccurrence (Figure 4C). Moreover, to investigate the oncogenic potential of hypoxia and its regulatory role in HIF2α-induced EFEMP1 expression in breast cancer, we analyzed the EFEMP1 expression levels in the presence or absence of HIF2α inhibitor 76 under CoCl_2_ treatment. Approximate IC_50_ values were determined using a dose-response curve (Supplementary Figure 2). Consistent with the results from microarray studies and qPCR validation, CoCl_2_-induced expression levels of EFEMP1 were significantly lower after HIF2α inhibitor 76 treatment in multiple breast cancer cell types (Figure 4D), suggesting that hypoxia-induced HIF2α expression is highly associated with enhanced EFEMP1 expression in breast cancer.

**Figure 5:**
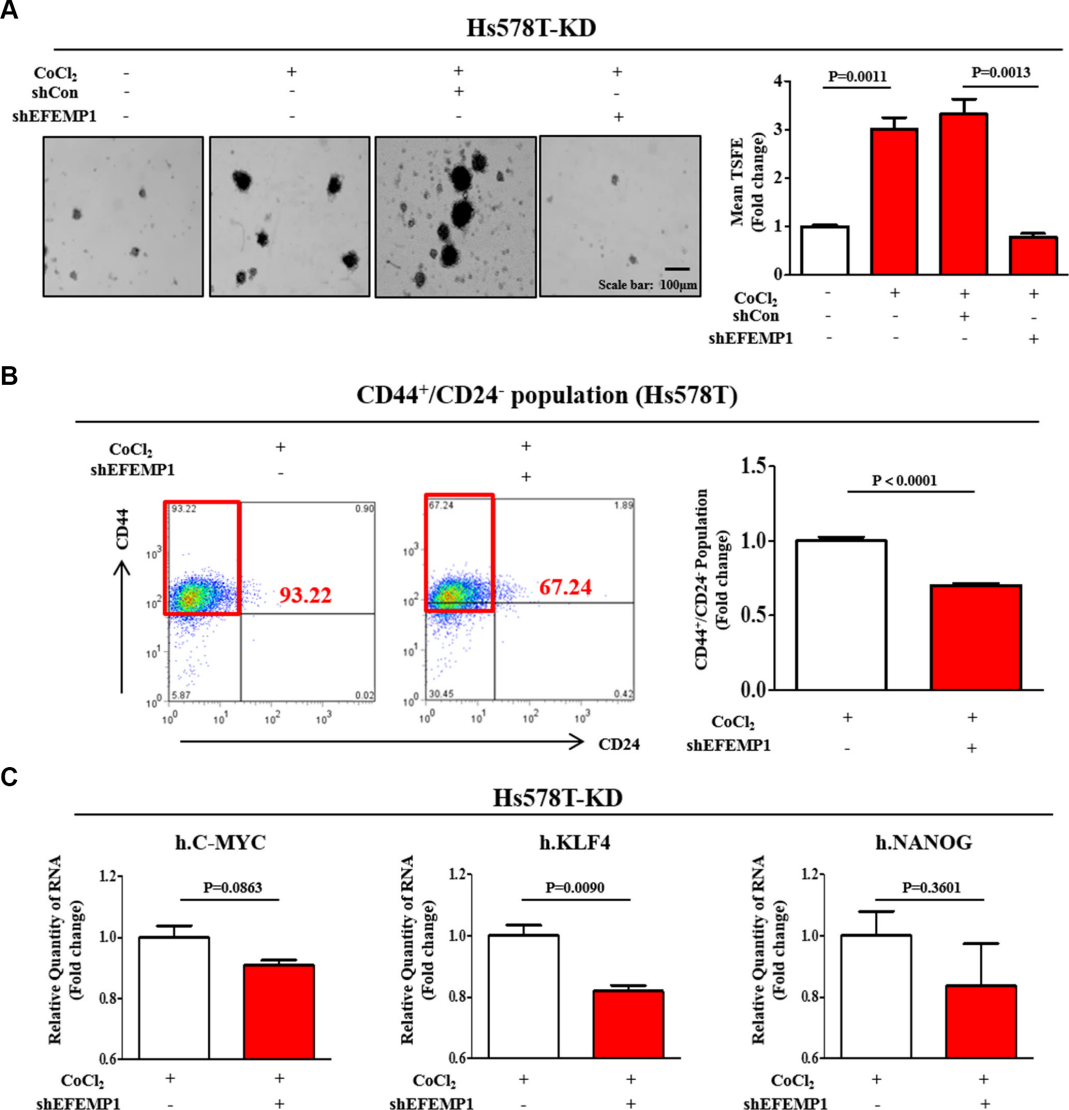
EFEMP1 knockdown suppressed CoCl_2_-induced growth and stemness-related features of BCSCs (**A**) Primary mammospheres from Hs578T cells treated with EFEMP1 shRNA under CoCl_2_ treatment were evaluated for the relative numbers of sphere-forming units. The stimulatory effects of hypoxia on BCSC sphere formation were successfully attenuated by EFEMP1 knockdown in Hs578T cells. (**B**) EFEMP1 knockdown led to a decrease in the percentage of CD44^+^/CD24^−^-positive cells as a proportion of total cancer cells under CoCl_2_treatment for 24h in Hs578T cells. (**C**) Hs578T cells treated with EFEMP1 shRNA were evaluated for the expression levels of stem cell markers c-Myc, Klf4, and Nanog by real-time PCR. Abbreviations: TSFE, Tumor sphere-forming efficiency. The results represent the mean ± SD from three independent experiments.

To determine whether EFEMP1 knockdown is sufficient to inhibit hypoxia-induced BCSC sphere formation, we knocked down EFEMP1 using specific shRNA in Hs578T cells. A stable cell line with EFEMP1 knock-down was established using a lentivirus system (Supplementary Figure 9A–9C). As a functional assay, we evaluated the effect of EFEMP1 knockdown on BCSC sphere formation. The stimulatory effects of CoCl_2_ on BCSC sphere formation were successfully attenuated by EFEMP1 knockdown in Hs578T cells (Figure 5A). Consistent with the results obtained from sphere formation, EFEMP1 knockdown led a significant decrease in CoCl_2_-induced CD44^+^/CD24^−^ subpopulations in Hs578T cells (Figure 5B). In this context, we examined the expression of BCSC markers with or without EFEMP1 knockdown in Hs578T cells. EFEMP1 knockdown moderately decreased the expression levels of stem cell markers c-Myc, Klf4, and Nanog under CoCl_2_ treatment (Figure 5C). These results suggest that HIF2α mediates CoCl_2_-induced cancer growth and that EFEMP1 is a downstream effector of hypoxia-induced HIF2α in breast tumorigenesis. Furthermore, we also examined the expression of EFEMP1 with or without HIF1α specific shRNA transection to further investigate whether HIF1α involved in the regulation of EFEMP1 expression. As expected, HIF1α knockdown does not affect the expression level of EFEMP1 in all three additional independent experiments (Supplementary Figure 10). These results suggest that HIF1α may be not involved in EFEMP1 expression.

### EFEMP1 knockdown reduces tumor growth in a murine xenograft model

**Figure 6:**
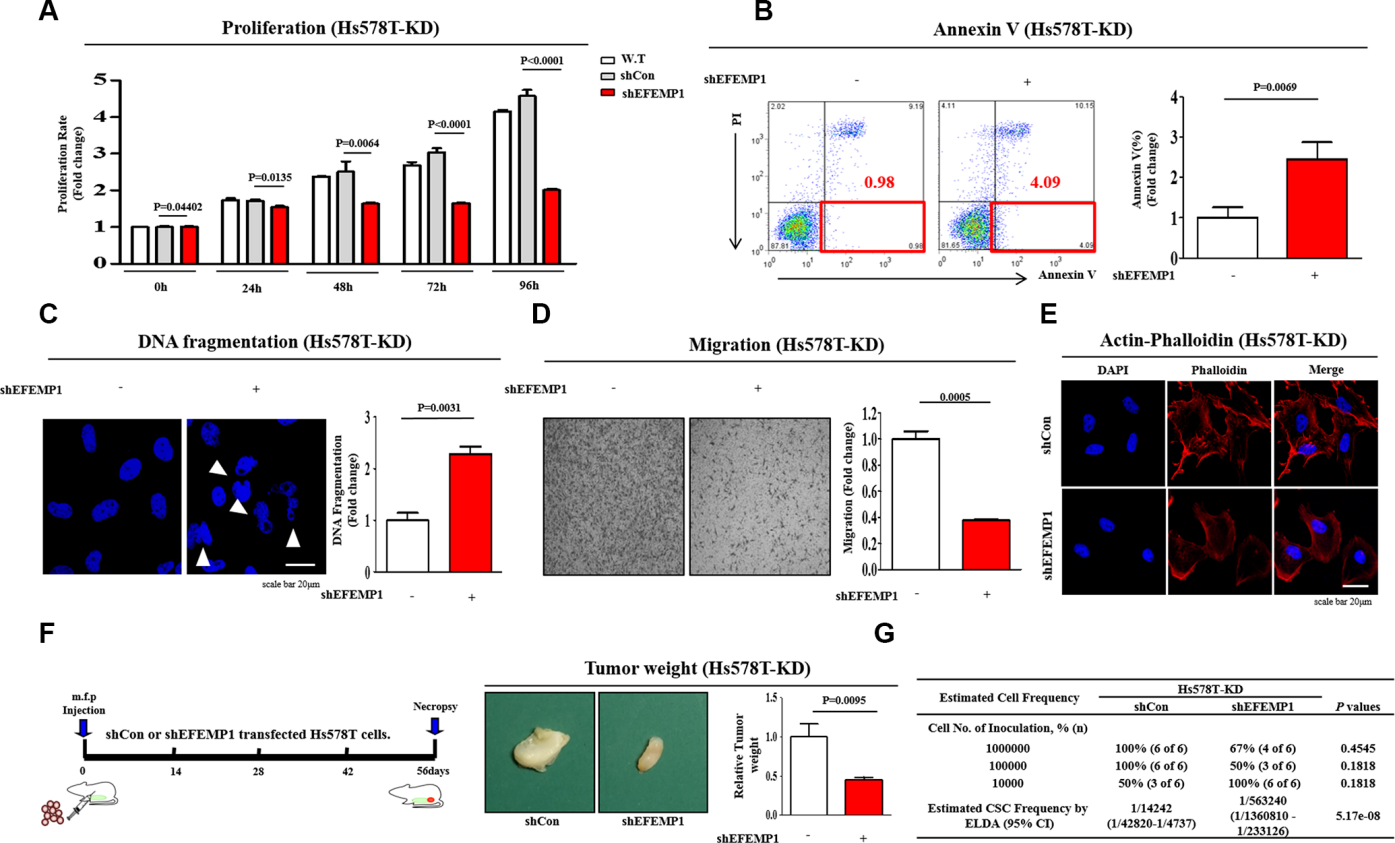
The effects of EFEMP1 knockdown on tumor growth, apoptosis and metastasis (**A**) Transfection of cells with EFEMP1 shRNA led to a time-dependent decrease in the number of cells compared with transfection using control shRNA. (**B**) EFEMP1 knockdown-mediated cytotoxicity was evaluated by flow cytometry using PE-labeled Annexin-V. (**C**) EFEMP1 knockdown-mediated apoptotic DNA fragmentation and condensation were visualized using DAPI staining. (**D**) Cell migration ability was evaluated using the transwell migration assay. Transfection with EFEMP1 shRNA significantly decreased Hs578T cell migration across the membrane compared with transfection using control shRNA. (**E**) EFEMP1 knockdown-induced fiber disorganization and a full morphological transition were visualized by actin-phalloidin staining. (**F**) Mice were implanted with Hs578T cells (1 × 10^6^ cells/mouse) by orthotopic injection into thoracic mammary fat pads. Tumor tissue was isolated from mice bearing Hs578T cells transfected with EFEMP1 shRNA or control shRNA. Tumor volumes were measured as described in the Materials and Methods section. (**G**) Hs578T cells transfected with EFEMP1 shRNA or control shRNA were injected into the mammary fat pads of mice in limiting dilutions (from 1 × 10^4^ to 1 × 10^6^ cells). Tumor formation was observed for 8 weeks following inoculation. BCSC frequency was calculated using an extreme limiting dilution assay (ELDA). The results are the mean ± SD from three independent experiments.

To assess the effect of EFEMP1 knockdown on breast cancer cell growth, cell viability was measured using an MTT assay. Briefly, cells were seeded in 96-well plates. After 48 h of incubation, cell viability was assessed by cell counting kit-8 (Dojindo Molecular Technologies Inc) according to the manufacturer's instruction. The numbers of viable cells were measured at a wavelength of 450 nm using Versamax microplate reader. As shown in Figure 6A, a time-dependent decrease in the number of EFEMP1-knockdown cells was observed compared with the control shRNA-infected cells. A flow cytometry assay using PE-labeled Annexin-V was used to evaluate the effect of EFEMP1 knockdown on apoptosis. The apoptotic rate of Hs578T cells transfected with EFEMP1-specific shRNA reached 7.48%, whereas this rate was 1.6% in control shRNA-transfected cells (Figure 6B). Apoptotic cell death was qualitatively estimated by DAPI staining for nuclear condensation and fragmentation. EFEMP1 knockdown led to significant DNA fragmentation compared with shRNA controls (Figure 6C). We also investigated the roles of EFEMP1 in the invasion and migration of human breast cancer cells using a transwell migration assay. Cells that migrated across the membrane were stained purple and counted. The results showed that following EFEMP1 knockdown, the migration ability across the transwell membrane was significantly decreased in the upper compartment of the transwell units (Figure 6D), suggesting that EFEMP1 is necessary for migration and might thus play a role in breast cancer metastasis. Previous studies have indicated that the actin cytoskeleton is required for tumor cell migration by pushing or pulling on the substrate near the cell membrane [33]. Therefore, we examined the distribution of the actin cytoskeleton at the subcellular level in Hs578T cells following EFEMP1 knockdown. Actin-phalloidin staining revealed a strong correlation between EFEMP1 knockdown and a highly disorganized actin cytoskeleton (Figure 6E), suggesting that the reduced migration of EFEMP1-knockdown cells may be related to the disorganization of the actin cytoskeleton.

Following our *in vitro* experiments, we further investigated the *in vivo* efficacy of EFEMP1 knockdown on tumorigenesis using a mouse xenograft model. EFEMP1-knockdown Hs578T cells were injected into the mammary fat pads of female nude mice, and tumor formation was monitored. Importantly, there was a consistent and significant reduction in tumor outgrowth in mice that were injected with EFEMP1 knockdown cells compared with control cells, indicating that EFEMP1 knockdown significantly impaired the tumor initiation potential of BCSCs (Figure 6F). We further performed an extreme limiting dilution assay (ELDA) to evaluate the inhibitory effect of EFEMP1 knockdown on tumorigenesis. Following the isolation of cells from freshly digested tumor tissues, we transplanted limiting dilutions (from 1 × 10^4^ to 1 × 10^6^ cells) of the cell preparations into mice. The repopulating unit frequency of the basal population was 1/4242 for controls and 1/563240 for EFEMP1 knockdown in Hs578T cells (Figure 6G).

## DISCUSSION

Approximately 30% to 50% of the patients diagnosed with early stage breast cancer are likely to progress to the metastatic stage, despite intensive conventional treatment, including surgery and chemotherapy [34]. Thus, the CSC concept has emerged as an important milestone in the understanding of chemodrug resistance and cancer recurrence [35]. Based on their characteristics, targeting and eradicating CSCs represents a potential strategy for significantly improving clinical outcomes. Moreover, Lagadec et al. revealed a significant increase in the BCSC population in various breast cancer cell lines after conventional chemo-drug treatment under hypoxic conditions [36]. Thus, the identification and development of new therapeutic approaches based on targeting the hypoxia-dependent responses in BCSCs is urgent.

In recent years, a number of studies have suggested that hypoxia appears to be strongly associated with various physiological processes, particularly when rapid tissue growth far exceeds blood supply. For example, rapidly growing tumors occur in a physiologic “hypoxic” environment (1%–2% O_2_) [37]. The hypoxic niche has been shown to play an important role in the maintenance of normal stem cells, but its roles in CSC function are largely unknown. Recently, our group identified hypoxia as one of the prominent micro-environmental factors regulating the self-renewal ability of BCSCs, partially by enhancing CSC phenotypes such as aldehyde dehydrogenase 1 (ALDH1) activity [28]. Consistent with the previous results, the present study shows that the number and size of CSC spheres, the expression levels of stem cell markers (c-Myc, Klf4, Oct4, and Nanog), and the percentage of cells with a CD44^+^/*CD24*^−/low^ phenotype were significantly increased by hypoxia in both mouse and human breast cancer cells (Figure 1A–1C).

The stimulatory effects of hypoxia on the self-renewal and tumor initiation of CSCs seem to be primarily mediated by HIFs, particularly HIF2α. Since its initial discovery, HIF2α has been demonstrated to have common transcriptional target genes with HIF1α, such as Ang2, Tie-2, and VEGF. Both HIF1α and HIF2α also show significant homology with the putative DNA binding sites [38]. Despite their significant similarities, HIF2α expression was principally restricted to most vascular endothelial cells [39] and had many different transcriptional target genes, including pluripotency-associated genes [11, 15]. These results suggest an important and unexpected role for HIF2α in the regulation of cancer progression, such as the tumorigenic potential of CSCs. Li et al. showed that HIF2α is co-localized with cancer stem cell markers in glioblastomas and its expression correlates with poor glioma patient survival [10]. This finding suggests a specific role for HIF2α in promoting self-renewal and stem-like CSC phenotypes. Pahlman et al. also demonstrated that high HIF-2α expression further correlates with more immature phenotypes and poor outcomes in patients undergoing brain tumor surgery [13]. Moreover, hypoxia-induced HIF2α also increases the expression of stem cell-associated genes (Oct4 [15] and c-Myc [11]) and confers tumorigenic potential to non-CSCs [14]. Because both Oct4 and c-Myc are factors modulating stem cell self-renewal ability and differentiation, these data shed light on how hypoxia-induced HIF2α stimulates the tumorigenic potential of BCSCs. Consistent with this hypothesis, our results showed that the stimulatory effects of hypoxia on BCSC sphere formation, CD44^+^/CD24^−/low^ subpopulations, and the expression levels of stem cell markers (c-Myc, Klf4, Oct4, and Nanog) were successfully attenuated by both HIF2α inhibitor 76 treatment and HIF2α knockdown (Figures 2 and 3). These findings are significant in showing that hypoxia-induced HIF2α may alter basic gene expression in breast cancer cells in a manner that can be transmitted to more stem-like phenotypes.

To further identify genes downstream of HIF2α under hypoxic conditions, we performed a microarray. Accordingly, our data show that EFEMP1 mRNA levels were significantly suppressed in HIF2α knockdowns compared with shRNA controls (Figure 4A). EFEMP1, which is also known as Fibulin-3, is a member of the fibulin family of extracellular matrix glycoproteins [16]. In cancer, multiple and diverse functions for fibulin family members have been suggested [40]. It has been shown that fibulin-1 mediates chemo-resistance in breast cancer cells and furthermore seems to play pivotal roles in cancer immuno-surveillance [19, 41]. Several previous studies have implied a potential role of the fibulin family members in colorectal tumorigenesis and epithelial-mesenchymal transition (EMT) in breast carcinoma [42, 43]. Interestingly, the role of HIF2α on EFEMP1 regulation is further supported by data from both HIF2α inhibitor 76 treatment and HIF2α knockdown experiments, in which HIF2α-depletion was correlated with decreased EFEMP1 levels (≥ 5.6 and 3.6-fold down-regulated, respectively) under hypoxic conditions (Figure 4B). More strikingly, our data indicate that the down-regulation of EFEMP1 expression is associated with the suppression of tumor growth and metastasis. The stimulatory effects of hypoxia on BCSC sphere formation were successfully attenuated by EFEMP1 knockdown in Hs578T cells (Figure 5A). Consistent with the results obtained from the sphere formation assay, EFEMP1 knockdown led to smaller CD44^+^/CD24^−/low^ subpopulations in Hs578T cells under hypoxic conditions (Figure 5B). Indeed, stable transfection of Hs578T cells with an shRNA construct targeting EFEMP1 results in a significant suppression of cell migration across the transwell membrane (Figure 6D), suggesting that EFEMP1 is necessary for migration and therefore might play roles in breast cancer metastasis.

Moreover, remodeling of the actin cytoskeleton is required for tumor cell migration because the cytoskeleton pushes or pulls on the substrate near the cell membrane [33], and such changes are induced by EFEMP1 knockdown (Figure 6E). These changes may result in accelerated metastasis and lead to greater tumorigenesis *in vivo*. Therefore, to further confirm the connection between EFEMP1 and breast cancer tumorigenesis *in vitro*, we assessed the effects of EFEMP1 knockdown on tumorigenesis using an *in vivo* Hs578T cell model. We found that the tumor formation in the mammary fat pads of female nude mice was significantly reduced in the EFEMP1-transfected groups compared with the control shRNA-infected groups in metastatic tumor models (Figure 6F and 6G), indicating that EFEMP1 depletion significantly impaired the tumor initiation potential of BCSCs. These findings are consistent with the potential importance of HIF2α/EFEMP1 cascades in breast cancer growth *in vitro*.

**Figure 7:**
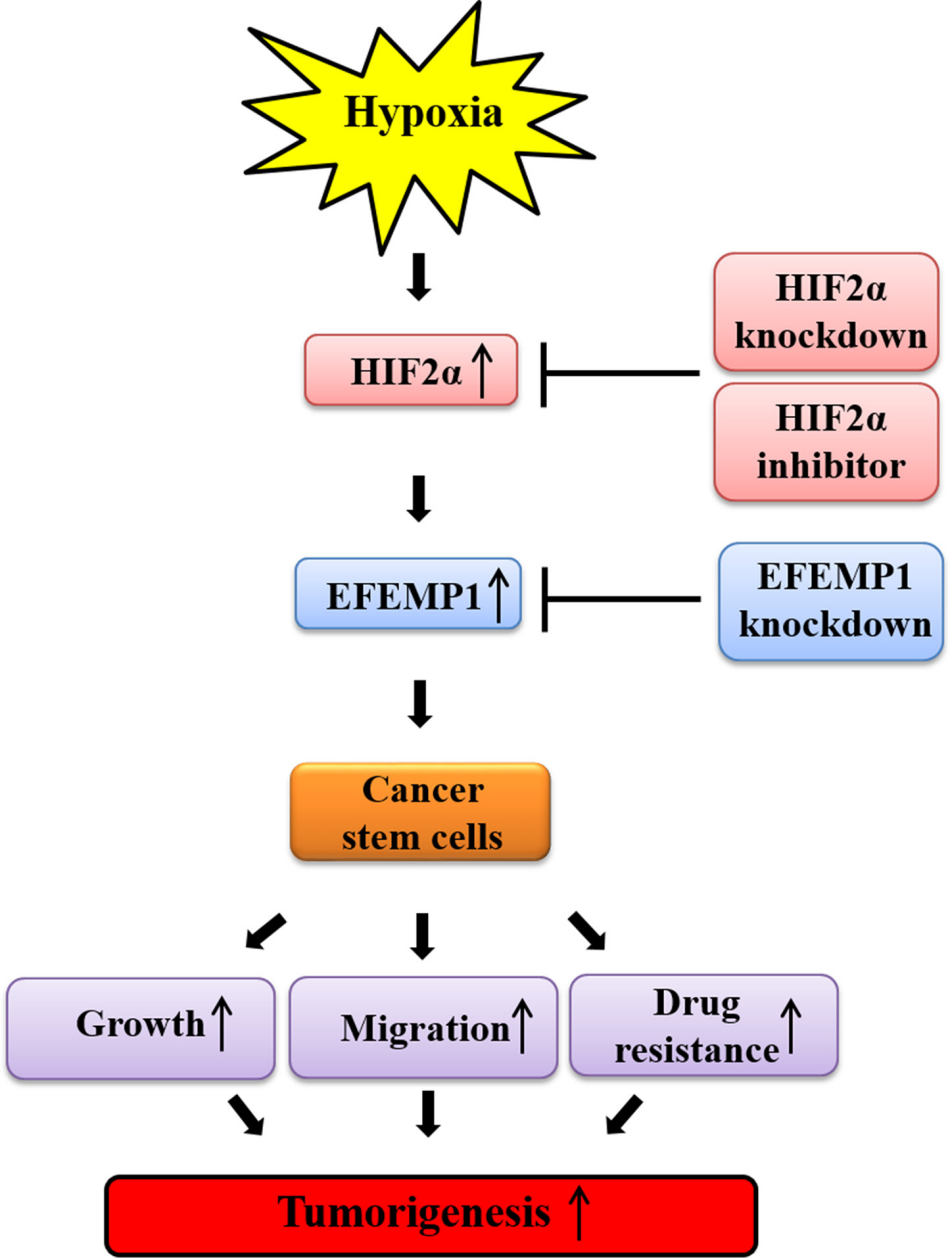
Schematic summary of the role of the HIF2α/EFEMP1 cascade in the development of metastatic breast cancer HIF2α/EFEMP1 cascade stimulates self-renewal and migration of CSCs, thereby promoting tumor growth and metastasis/systemic dissemination in breast cancer.

In summary, consistent with previous studies [44], our study demonstrates that the stimulatory effects of hypoxia on BCSC sphere formation, CD44^+^/CD24^−^ subpopulations, and the expression levels of stem cell markers (c-Myc, Klf4, Oct4, and Nanog) were successfully attenuated by HIF2α-depletion. These findings suggest that the inhibition of hypoxia-induced HIF2α suppresses the growth and functionality of BCSCs, which might be key drivers of breast cancer metastasis and recurrence. To the best of our knowledge, the stimulatory effects of hypoxia-induced HIF2α on BCSC sphere formation were successfully attenuated by EFEMP1 knockdown in Hs578T cells. Taken together, these data suggest that HIF2α, but not HIF1α, mediates hypoxia-induced cancer growth/metastasis and that EFEMP1 is a downstream effector of hypoxia-induced HIF2α during breast tumorigenesis, as illustrated in Figure 7.

## MATERIALS AND METHODS

### Cell culture and reagents

Murine mammary cancer cell line 4T1 and human breast cancer cell line Hs578T were cultured in DMEM (Invitrogen, Grand Island, NY) supplemented with 10% fetal bovine serum (FBS), 100 U/ml penicillin and 100 U/ml streptomycin (Lonza, Basel, Switzerland) at 37°C and 5% CO_2_. HIF2α inhibitor 76 purchased from Calbiochem (San Diego, CA).

### Short hairpin RNA

Small hairpin RNA (shRNA) targeting mouse HIF2α (TRCN0000003806, NM_001430X-1694S1C1), EFEMP1 (TRCN00000055963, NM_004105.2-372S1C1), and non-targeting RNA (SHC001) were purchased from Sigma (St. Louis, MO, USA). For the efficient HIF2α and EFEMP1 shRNA transfection, transfection was performed using Lipofectamine 2000 (Invitrogen) according to the manufacturer's instructions. We chose the HIF2α and EFEMP1 shRNA that is most effective in mRNA levels from five shRNA designed from the target sequence and determined by qRT-PCR.

### Tumorsphere formation

Single cells were resuspended in serum-free DMEM (Invitrogen) containing B27 (Invitrogen), 20 ng/ml EGF, 20 ng/ml bFGF (PeproTech) and 4 μg/ml heparin (Sigma-Aldrich). Primary tumorspheres were derived by plating 50,000 single cells/well into six-well ultra-low attachment dishes (Corning). Individual spheres ≥ 100 μm from each replicate well (*n* ≥ 9 wells) were counted under an inverted microscope at 50× magnification using the Image-Pro Plus program (Media Cybernetics). The percentage of cells capable of forming spheres, termed the ‘tumorsphere formation efficiency (TSFE)’, was calculated as follows: [(number of sphere formed/number of single cells plated) × 100].

### Cell proliferation assay

4T1 and Hs578T cells were seeded in 96-well plates. After 48 h of incubation, cell viability was assessed by cell counting kit-8 (Dojindo) according to the manufacturer's instruction. The numbers of viable cells were measured at a wavelength of 450 nm using Versamax microplate reader.

### Real-time PCR and Microarray

Total RNA was extracted using TRIzol reagent (Invitrogen). RNA purity was verified by measuring 260/280 absorbance ratio. The first strand of cDNA was synthesized with 1μg of total RNA using SuperScript II (Invitrogen), and one-tenth of the cDNA was used for each PCR mixture containing Express SYBR-Green qPCR Supermix (BioPrince, Seoul, Korea). Real-time PCR was performed using a Rotor-Gene Q (Qiagen). The reaction was subjected to 40-cycle amplification at 95°C for 20 sec, at 60°C for 20 sec and at 72°C for 25 sec. Relative mRNA expression of selected genes was normalized to HPRT and quantified using the ΔΔCT method (ΔΔCt: target/reference ratio in experimental sample relative to target/reference ratio). The sequences of the PCR primers are listed in Table 1. Microarray array was performed whole genome gene-expression analysis using GeneChip^®^ Human Gene 1.0 ST Array from DNA Link USA, Inc. The microarray array was analyzed using Ingenuity Pathway Analysis (IPA) software. The genes whose expression decreased more than 1.5-fold are listed in Supplementary Table 1.

**Table 1:** Primer sequences quantitative RT-PCR

Gene	Genebank No.	Direction	Primer sequence
Mouse C-myc	NM_010849	F	CGGACACACAACGTCTTGGAA
		R	AGGATGTAGGCGGTGGCTTTT
Mouse Klf4	NM_010637	F	GGTGCAGCTTGCAGCAGTAA
		R	AAAGTCTAGGTCCAGGAGGTCGTT
Mouse Oct4	NM_013633	F	GCATTCAAACTGAGGCACCA
		R	AGCTTCTTTCCCCATCCCA
Mouse Nanog	NM_028016	F	GCCTTACGTACAGTT GCAGCAA
		R	TCACCTGGTGGAGTCACAGAGT
Mouse EFEMP1	NM_146015	F	AACTATGCTGACTCTGGCGCTG
		R	ATCCATCGGTGCATTGCGT
Mouse HPRT	NM_013556	F	GCCTAAGATGAGCGCAAGTTG
		R	TACTAGGCAGATGGCCACAGG
Human C-MYC	NM_002467	F	AAAGGCCCCCAAGGTAGTTA
		R	GCACAAGAGTTCCGTAGCTG
Human KLF4	NM_004235	F	GAACTGACCAGGCACTACCG
		R	TTCTGGCAGTGTGGGTCATA
Human OCT4	NM_002701	F	ACATCAAAGCTCTGCAGAAAGAACT
		R	CTGAATACCTTCCCAAATAGAACCC
Human NANOG	NM_024865	F	ACATGCAACCTGAAGACGTGTG
		R	CATGGAAACCAGAACACGTGG
Human EFEMP1	NM_004105	F	GTGCACTGCAGGGACGCACA
		R	CGCACTGCTCCCCTCGCTTC
Human PPIA	NM_021130	F	TGCCATCGCCAAGGAGTAG
		R	TGCACAGACGGTCACTCAAA

### Flow cytometry

FACS analysis and cell sorting were performed using FACS Calibur and FACS Aria machines (Becton Dickinson, Palo Alto, CA), respectively. FACS data were analyzed using Flowjo software (Tree Star, Ashland, OR). Antibodies to the following proteins were used: PE-conjugated CD44 (BD Bioscience, Cat.559942, dilution 1/40) and CD24 (BD Bioscience, Cat.555428, dilution 1/40). The FACS gates were established by staining with isotype antibody or secondary antibody.

### Protein isolation and western blot analysis

Protein expression levels were determined by western blot analysis as previously described. [45] Briefly, cells were lysed in a buffer containing 50 mM Tris, 5 mM EDTA, 150 mM NaCl, 1 mM DTT, 0.01 % NP 40, 0.2 mM PMSF. The protein concentrations of the total cell lysates were measured by using bovine serum albumin as standard. Samples containing equal amounts of protein were separated by sodium dodecyl sulfate polyacrylamide gel electrophoresis (SDS-PAGE) and then transferred onto polyvinylidene difluoride (PVDF) membranes (Bio-RAD Laboratories). The membranes were blocked with 5 % skim milk in Tris buffered saline containing Tween-20 at RT, and the membranes were with HIF1α (Abcam AB2185), HIF2α (Novus biologicals NB100-132), or EFEMP1(Santa Cruz Biotechnology, SC-365224) antibodies overnight at 4°C and then with HRP-conjugated secondary antibodies for 90 min at RT. Antibody-bound proteins were detected using an ECL.

### Immunofluorescent staining

Samples were fixed with 4% paraformaldehyde for fluorescent staining. Samples were permeabilized with 0.3 M glycine and 0.3% Triton X-100, and nonspecific binding was blocked with 2% normal swine serum (DAKO, Glostrup, Denmark). Staining was performed as described previously [46], using the primary anti-Phalloidin (Cytoskeleton Inc.) antibody. Samples were examined by fluorescence microscopy (Zeiss LSM 510 Meta). The calculation of expression was based on green fluorescence area and density divided by cell number, as determined from the number of DAPI-stained nuclei, in three randomly selected fields for each sample from a total of three independent experiments.

### *In vitro* cell migration assay

Cell were plated at 1 × 10^5^ cells/well in 200 μL of culture medium in the upper chamber of Transwell permeable supports (Corning Inc, Corning, NY) with 8.0- μm pore, polycarbonate membrane, 6.5-mm diameter, and 24-well plate format) to track migration of Hs578T cells. The cells on the upper surface of the membranes were completely removed by using a cotton swab. Migrated cells on the lower surface of the membranes were fixed with 4% paraformaldehyde for 10 min, stained with hematoxylin (Sigma-Aldrich), and later the number of cells was counted in three randomly selected fields of the wells under light microscope. To calculate the chemotactic index, the number of cells migrated in response to EFEMP1 knockdown was divided by the number of spontaneously migrated cells (control).

### Tumorigenesis experiment

All animal experiments were approved and carried out in accordance with IACUC (Institutional Animal Care and Use Committee) guidelines (No.LCDI-2012-0069) of the Lee Gil Ya Cancer and Diabetes Institute. For tumorigenesis experiments, EFEMP1-knockdowned (1 × 10^6^ cells/mouse) or control (1 × 10^6^ cells/mouse) Hs578T cells were injected into the mammary fat pads of female nude mice in 50μL volume (*n* = 6 for each group), and tumor formation was measured after 8 weeks.

### *In vivo* extreme limiting dilution assay

For the limiting dilution experiment, primary tumors were minced using scissors and incubated in DMEM (Invitrogen) mixed with collagenase/hyaluronidase (Stem cell Technologies) at 37°C for 15-20 min. Primary tumor-derived cells were inoculated into the m.f.p. of mice at varying cell densities ranging from 1 × 10^4^ to 1 × 10^6^ cells in a total volume of 50 μL volume (*n* = 6 for each group). Hs578T cells-injected mice were euthanized on eight weeks. The frequency of tumor-initiating cells (TICs) was calculated using ELDA webtool (http://bioinf.wehi.edu.au/software/elda).

### Statistical analysis

All the statistical data were analyzed by GraphPad Prism 5.0 (GraphPad Software, San Diego, CA) and evaluated by two-tailed Student's *t*-test. Value of *P* < 0.05 was considered to indicate statistical significance.

## SUPPLEMENTARY MATERIALS FIGURES AND TABLE




