# Supplementary material for: HIF2α/EFEMP1 cascade mediates hypoxic effects on breast cancer stem cell hierarchy
Source: Oncotarget. 2016 Jun 6;7(28):43518–33. doi: 10.18632/oncotarget.9846 (PMC5190041; doi:10.18632/oncotarget.9846)
Supplement: Supplementary file 1 [file oncotarget-07-43518-s001.pdf]

HIF2α/EFEMP1 cascade mediates hypoxic effects on breast cancer stem cell hierarchy

Supplementary Materials

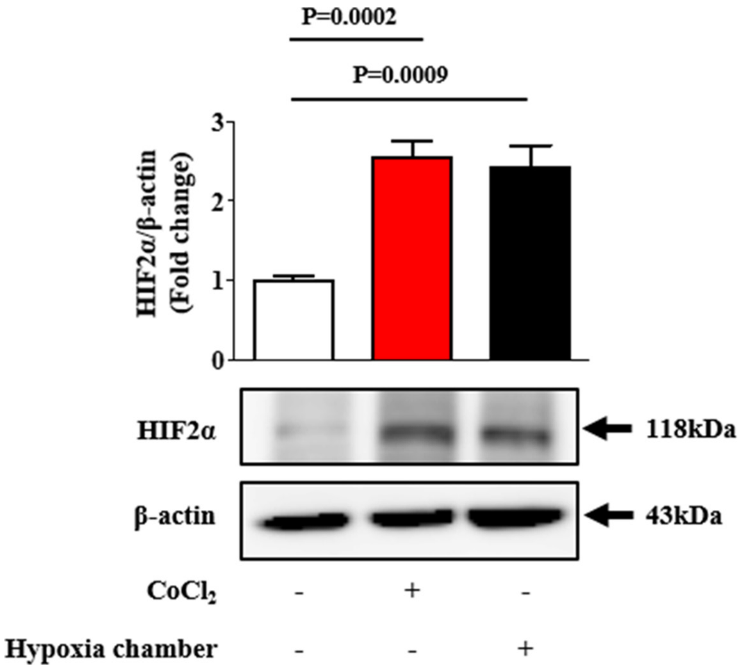

Supplementary Figure S1: The expression levels of HIF2α with or without CoCl2 treatment or hypoxic exposure. The effect of $CoCl^2$ (100 μM) and hypoxic exposure expression of HIF2α was assessed in 4T1 cells by western blot analysis. β-actin was used as the internal control. The results represent the mean ± SD from three independent experiments.

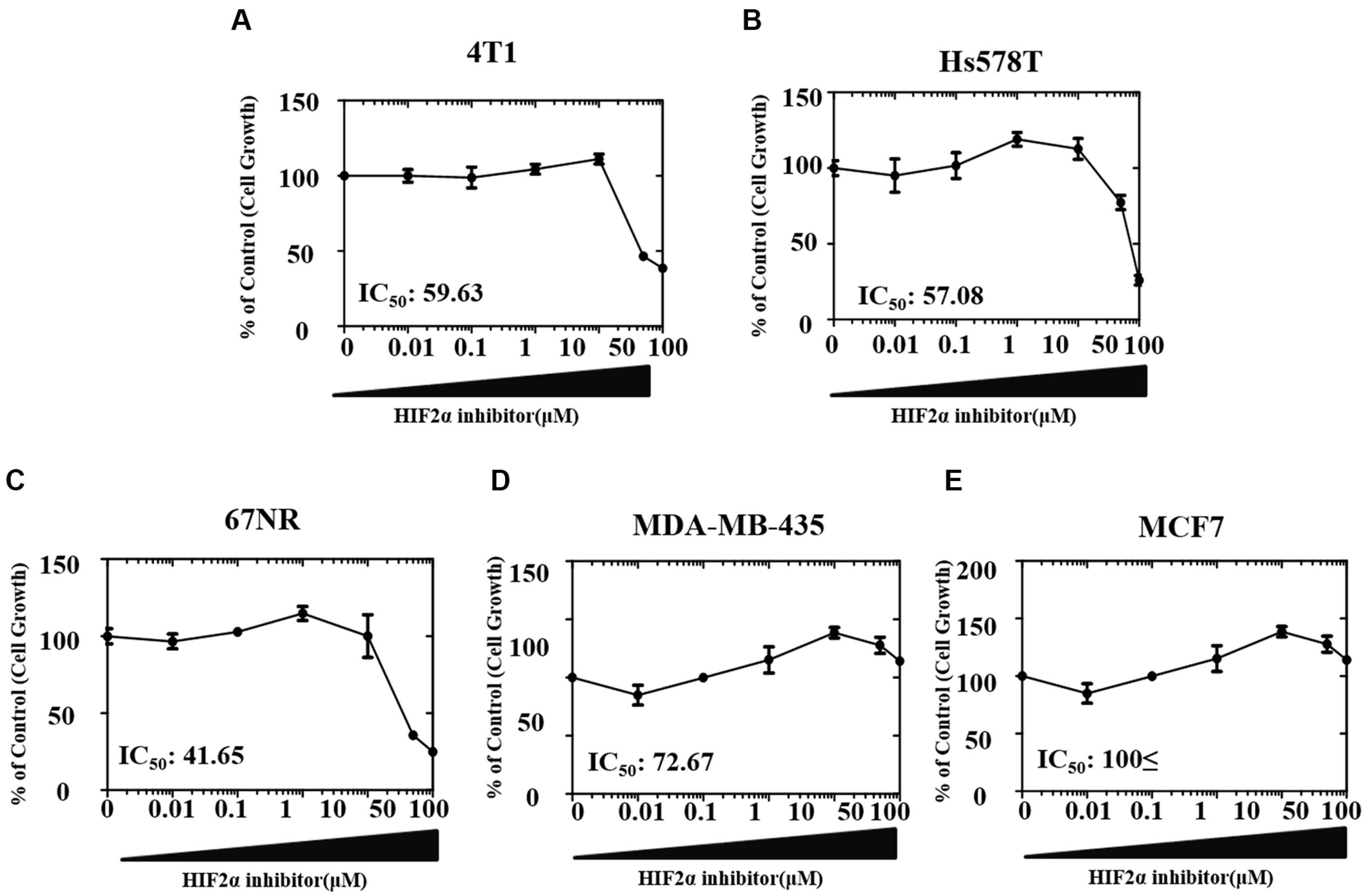

Supplementary Figure S2: The treatment concentration that inhibits 50% of the cell proliferation (IC_{50}). The inhibition of cell viability through HIF2α inhibitor 76 treatment for 48 hours was determined using a CCK-8 assay (mitochondrial dehydrogenase activity) in 4T1 (**A**), Hs578T (**B**), 67NR (**C**), MDA-MB-435 (**D**), and MCF-7 cells (**E**). The cell viability (%) was calculated as a percent of the vehicle control.

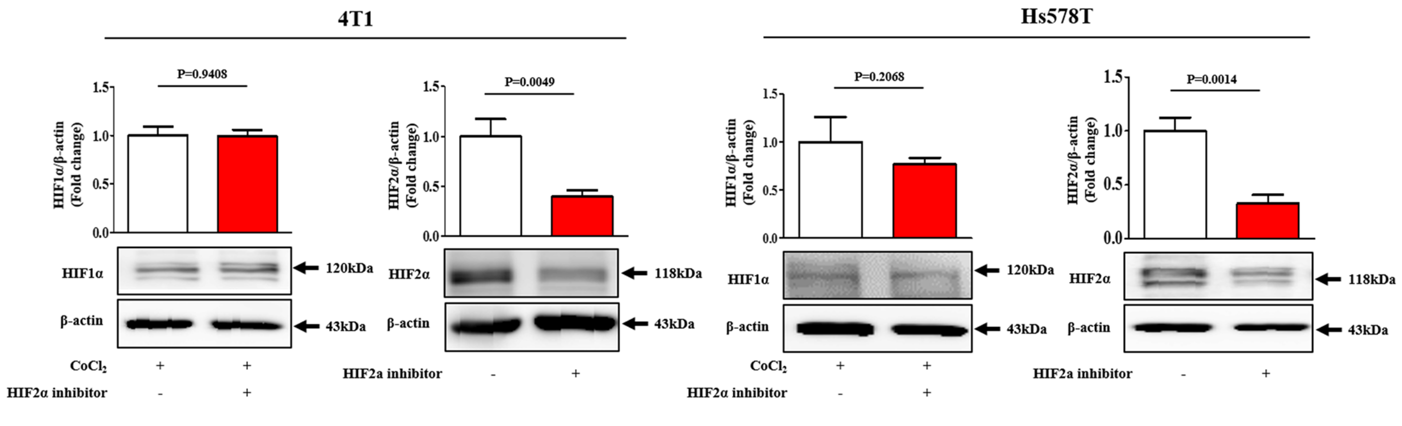

Supplementary Figure S3: The stimulatory effects of $CoCl_2$ on HIF1α expression were not affected by HIF2α-specific inhibitor treatment. The effects of small molecule HIF2α inhibitor 76 (10 μM for 4T1 cells; 25 μM for Hs578T cells) on $CoCl_2$ (100 μM)-induced expression of HIF1α and HIF2α were assessed in both 4T1 and Hs578T cells by western blot analysis. β-actin was used as the internal control. The results represent the mean ± SD from three independent experiments.

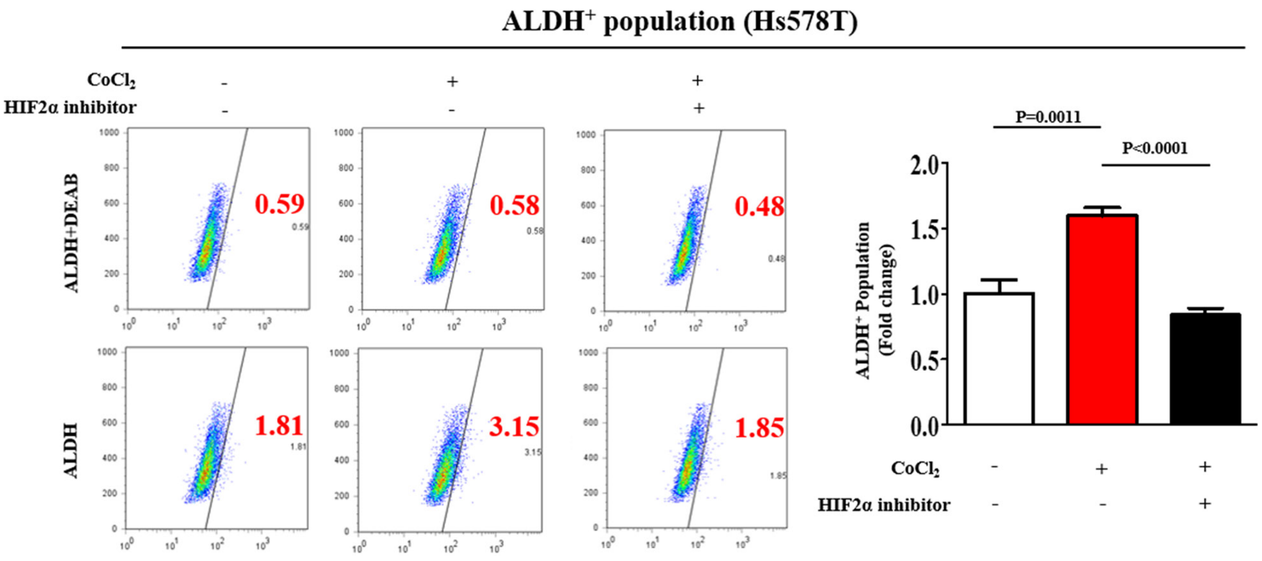

Supplementary Figure S4: HIF2α inhibitor 76 suppressed CoCl^2-enriched ALDH$^+$ subpopulation. The results of FACS analysis showing the percentage of the total cell population that consisted of ALDH$^+$ cells in Hs578T cells. $CoCl_2$ (100 μM) treatments led to an increase in the percentage of ALDH$^+$ cells as a proportion of the total cancer cells in Hs578T cells. The stimulatory effects of $CoCl_2$ on ALDH$^+$ subpopulation were successfully attenuated by the treatment of small molecule HIF2α inhibitor 76 (25 μM) in Hs578T cells. The results represent the mean ± SD from three independent experiments.

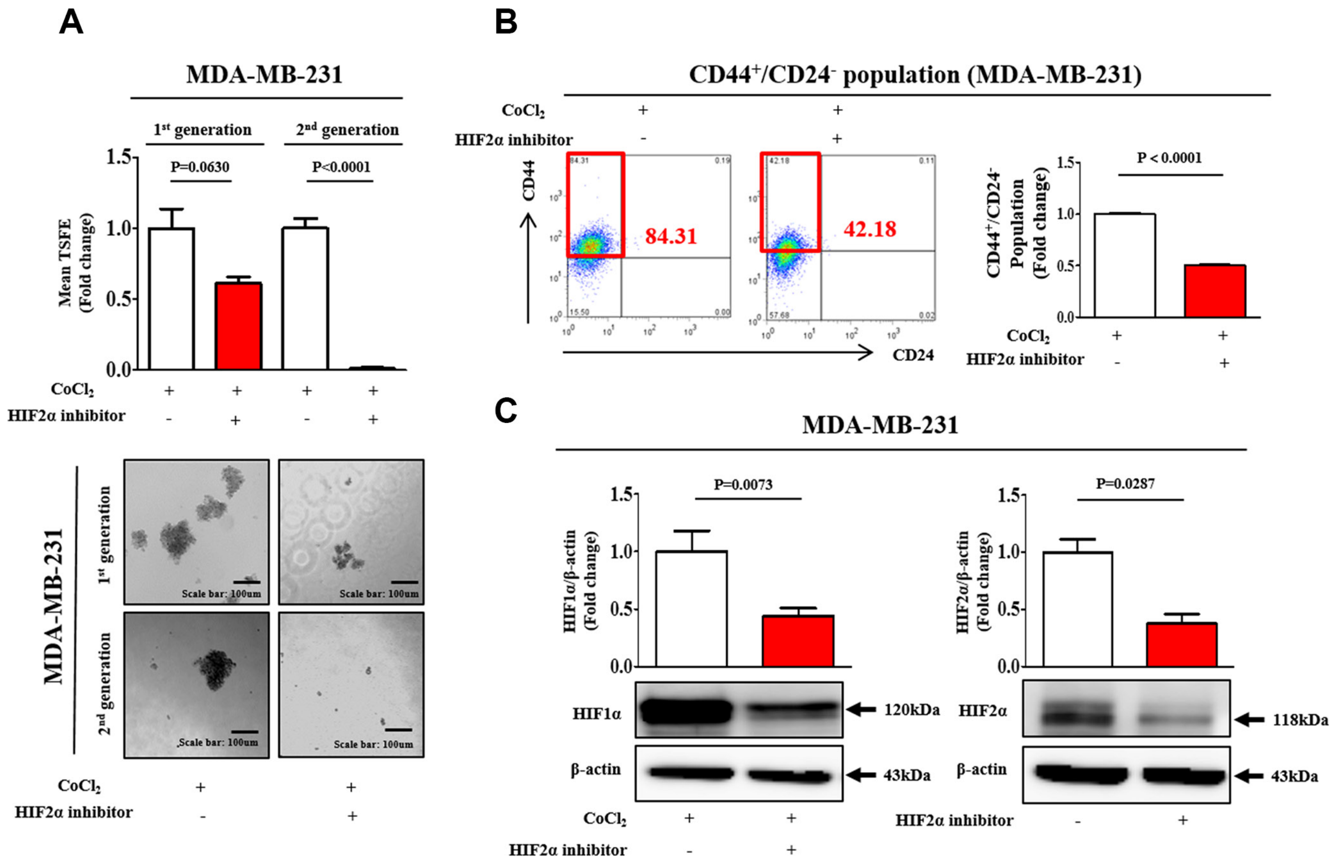

Supplementary Figure S5: HIF2α inhibitor 76 suppresses CoCl^2-enriched BCSC growth in MDA-MB-231 cells. (**A**) HIF2α inhibitor 76 inhibited $CoCl_2$ (100 μM)-induced primary (with HIF2α inhibitor 76 treatment) and second sphere formation (without additional HIF2α inhibitor 76 treatment) in MDA-MB-231 cells. (**B**) The results of FACS analysis showing the percentage of the total cell population that consisted of CD44^{+}/CD24^{-} cells in MDA-MB-231 cells. $CoCl_2$ (100 μM) treatments led to an increase in the percentage of CD44$^+$/CD24$^-$ cells as a proportion of the total cancer cells in MDA-MB-231 cells. The stimulatory effects of $CoCl_2$ on CD44$^+$/CD24$^-$ subpopulation were successfully attenuated by the treatment of small molecule HIF2α inhibitor 76 (25 μM) in MDA-MB-231 cells. The results represent the mean ± SD from three independent experiments. (**C**) The expression of HIF1α and 2α upon hypoxia with or without HIF2α specific shRNA transection were assessed in MDA-MB-231 cells by western blot analysis. Abbreviations: TSFE, Tumor sphere-forming efficiency. β-actin was used as the internal control. The results represent the mean ± SD from three independent experiments.

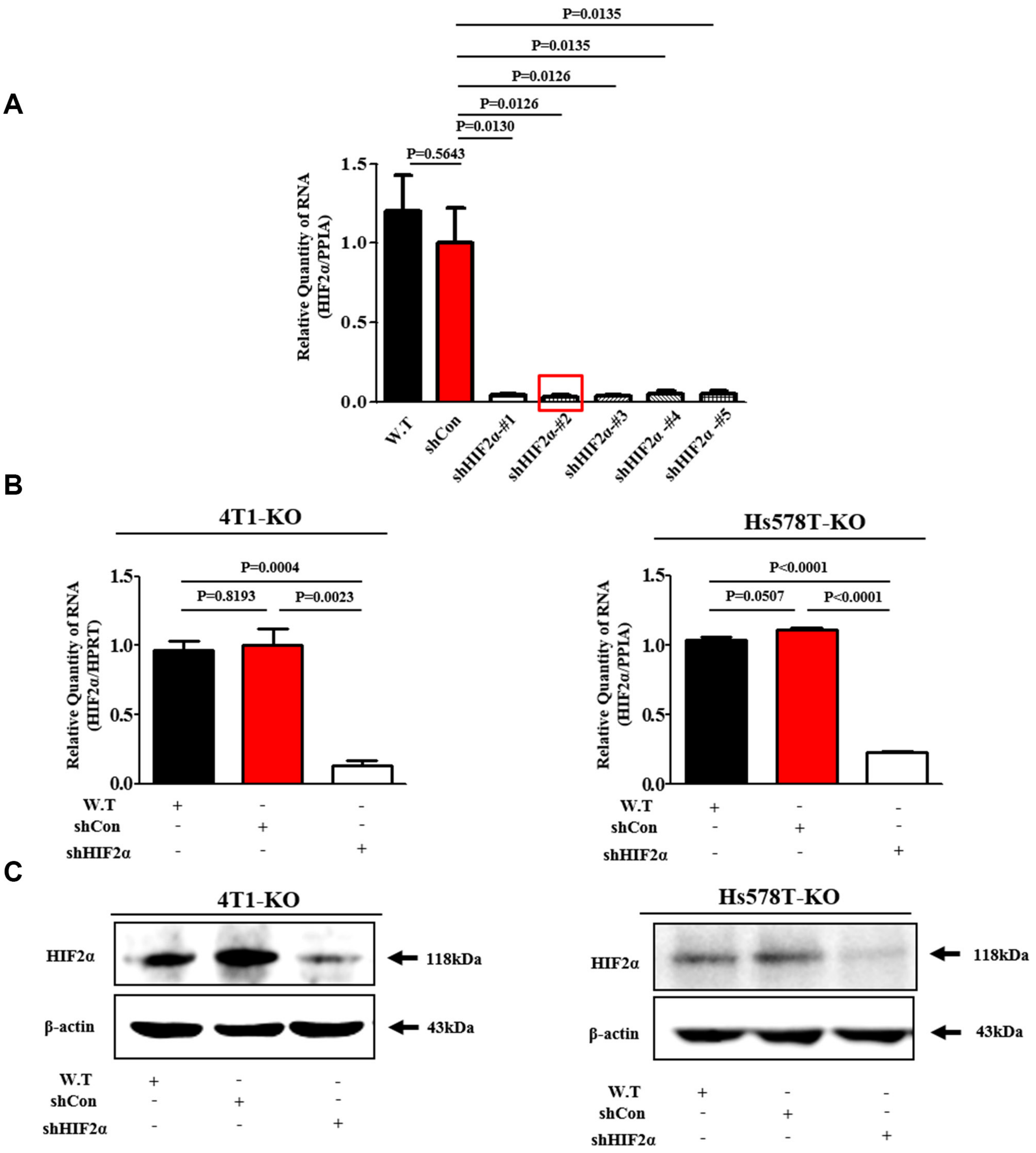

Supplementary Figure S6: Knockdown efficiency of shRNAs targeting HIF2α. (**A**) Both 4T1 and Hs578T cells were stably transduced with shRNA #1, #2, #3, #4, or #5 targeting HIF2α or with a non-targeting control shRNA. HIF2α shRNA construct #2, hereafter referred to as HIF2α shRNA, was the most efficient at knockdown. Successful knockdown of HIF2α was verified based on RNA (**B**) and protein levels (**C**) in both 4T1 and Hs578T cells. β-actin was used as the internal control. The results represent the mean ± SD from three independent experiments.

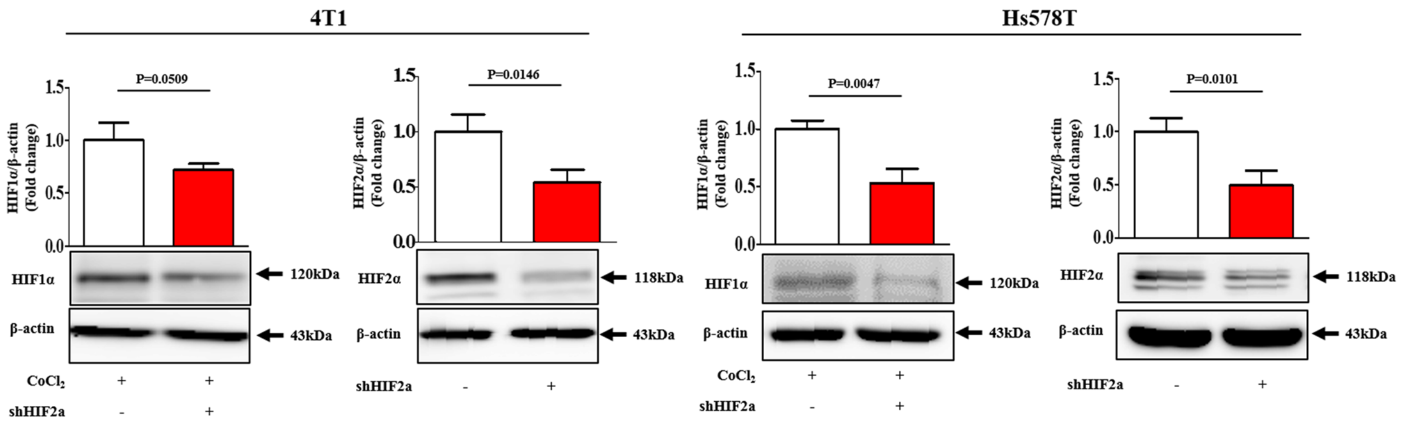

Supplementary Figure S7: The stimulatory effects of $CoCl_2$ on HIF1α expression were not affected by HIF2α Knockdown. The effects of HIF2α knockdown on $CoCl_2$ (100 μM)-induced expression of HIF1α and HIF2α were assessed in both 4T1 and Hs578T cells by western blot analysis. β-actin was used as the internal control. The results represent the mean ± SD from three independent experiments.

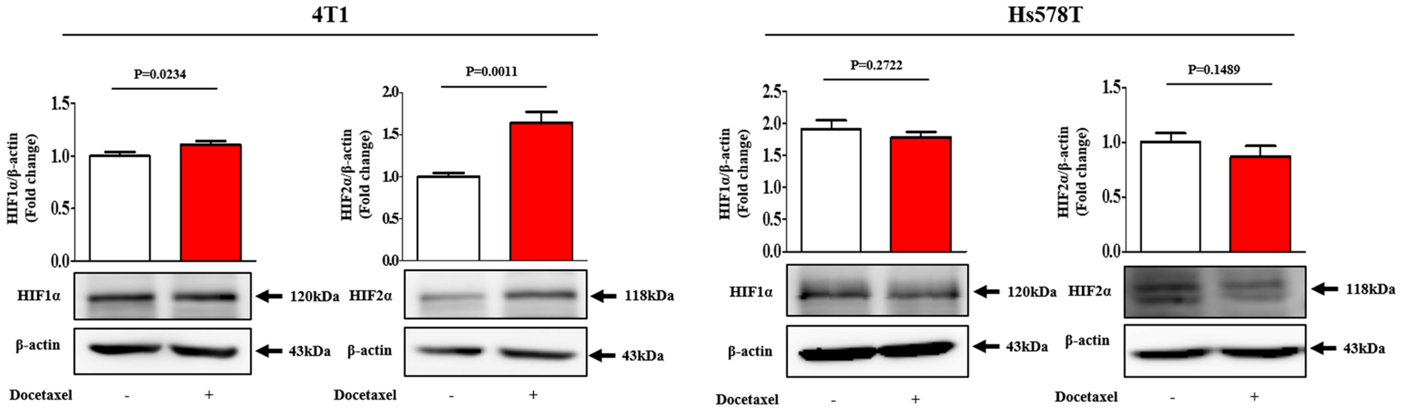

Supplementary Figure S8: The effects of docetaxel treatment in the expressions of HIF1α and HIF2α. Both 4T1 and Hs578T cells were treated with docetaxel (5 nM) and the expression of HIF1α and HIF2α were evaluated by western blot analysis. β-actin was used as the internal control. The results represent the mean ± SD from three independent experiments.

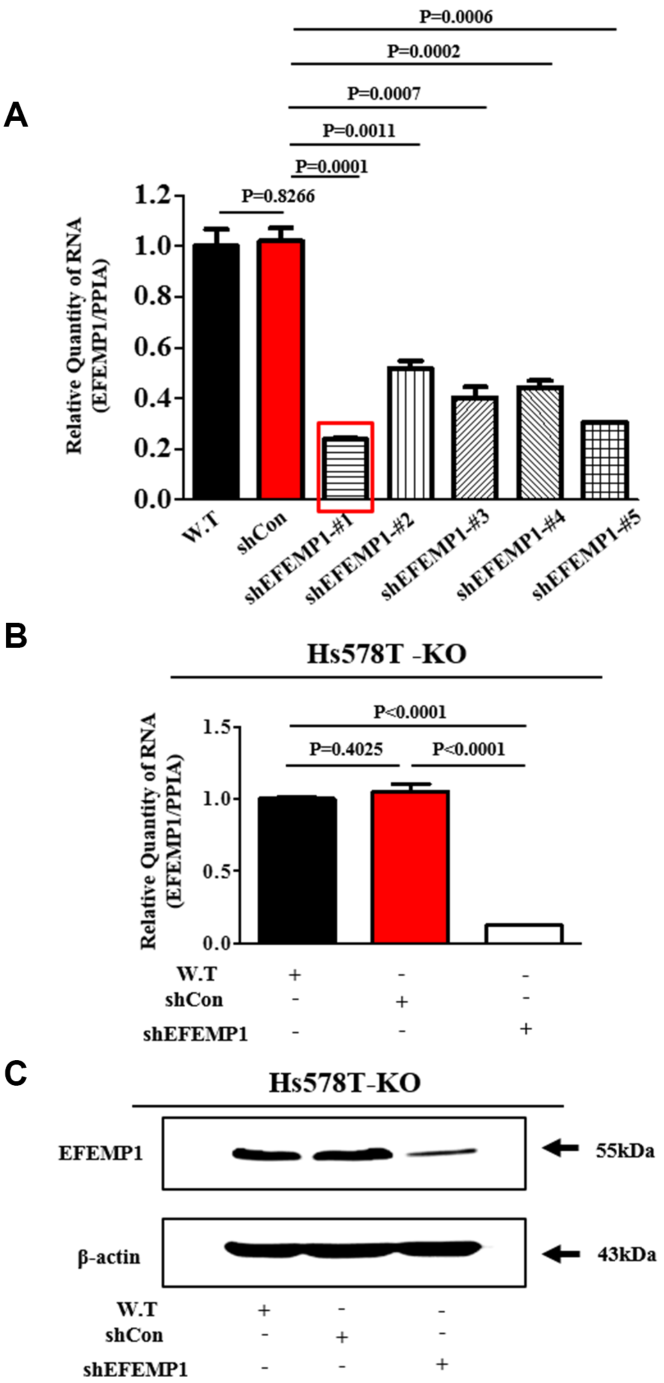

Supplementary Figure S9: Knockdown efficiency of shRNAs targeting EFEMP1. (**A**) Hs578T cells were stably transduced with shRNA #1, #2, #3, #4, or #5 targeting EFEMP1 or with a non-targeting control shRNA. EFEMP1 shRNA construct #1, hereafter referred to as EFEMP1 shRNA, was the most efficient at knockdown. Successful knockdown of EFEMP1 was verified based on RNA (**B**) and protein levels (**C**) in Hs578T cells. β-actin was used as the internal control. The results represent the mean ± SD from three independent experiments.

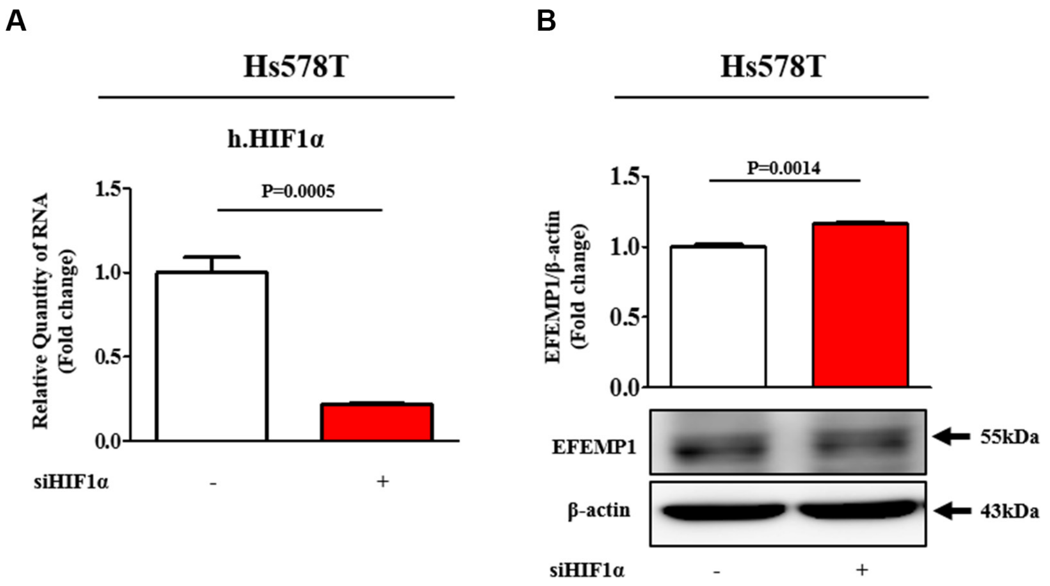

Supplementary Figure S10: The expression of EFEMP1 with or without HIF1α specific shRNA transection. (**A**) Hs578T cells were treated with HIF1α specific siRNA and (**B**) EFEMP1 expression were evaluated by western blot analysis. β-actin was used as the internal control. The results represent the mean ± SD from three independent experiments.

Supplementary Table S1: The list of genes which decreased more than 1.5-fold. See Supplementary_ Table_S1
