# Supplementary material for: HIF2α/EFEMP1 cascade mediates hypoxic effects on breast cancer stem cell hierarchy
Source: Oncotarget. 2016 Jun 6;7(28):43518–33. doi: 10.18632/oncotarget.9846 (PMC5190041; doi:10.18632/oncotarget.9846)
Supplement: Supplementary file 2 [file oncotarget-07-43518-s002.docx]

Supplementary Table S1: The list of genes which decreased more than 1.5-fold

| **Gene Symbol** | **Gene Description** | **average log2 ratio** | **absolute fold change** | **Gene Accession** |
| --- | --- | --- | --- | --- |
| ETV4 | ets variant 4 | –0.855812667 | –1.809777912 | NM_001079675 |
| OTUB2 | OTU domain, ubiquitin aldehyde binding 2 | –0.858402667 | –1.813029836 | NM_023112 |
| GPR125 | G protein–coupled receptor 125 | –0.864681333 | –1.820937409 | NM_145290 |
| FGD4 | FYVE, RhoGEF and PH domain containing 4 | –0.871609667 | –1.829703228 | NM_139241 |
| SYT16 | synaptotagmin XVI | –0.873027333 | –1.831502072 | NM_031914 |
| ERAP2 | endoplasmic reticulum aminopeptidase 2 | –0.882558 | –1.843641311 | NM_022350 |
| RAC2 | ras-related C3 botulinum toxin substrate 2 (rho family, small GTP binding protein Rac2) | –0.888908333 | –1.851774382 | NM_002872 |
| RHOJ | ras homolog gene family, member J | –0.889297667 | –1.852274179 | NM_020663 |
| ESM1 | endothelial cell-specific molecule 1 | –0.892676667 | –1.856617557 | NM_007036 |
| PTPN22 | protein tyrosine phosphatase, non-receptor type 22 (lymphoid) | –0.893067667 | –1.857120807 | NM_015967 |
| MCTP1 | multiple C2 domains, transmembrane 1 | –0.924723333 | –1.898320165 | NM_024717 |
| LIPG | lipase, endothelial | –0.945022333 | –1.925218689 | NM_006033 |
| HAX1 | HCLS1 associated protein X-1 | –0.95656 | –1.940676972 | NM_006118 |
| ANKRD20B | ankyrin repeat domain 20B | –0.956696667 | –1.940860822 | NR_003366 |
| TIAM1 | T-cell lymphoma invasion and metastasis 1 | –0.962594333 | –1.948811205 | NM_003253 |
| LMLN | leishmanolysin-like (metallopeptidase M8 family) | –0.965286 | –1.952450537 | NM_001136049 |
| FLJ45950 | FLJ45950 protein | –0.969880333 | –1.958678123 | AK127847 |
| FAM49A | family with sequence similarity 49, member A | –0.973207 | –1.96319979 | NM_030797 |
| AP1S3 | adaptor-related protein complex 1, sigma 3 subunit | –0.981473667 | –1.974481248 | NM_001039569 |
| MLKL | mixed lineage kinase domain-like | –0.982812667 | –1.976314663 | NM_152649 |
| EXPH5 | exophilin 5 | –1.015530667 | –2.021646379 | NM_015065 |
| SYT14 | synaptotagmin XIV | –1.019280333 | –2.026907615 | NR_027458 |
| TNFRSF10B | tumor necrosis factor receptor superfamily, member 10b | –1.0287 | –2.04018503 | NM_003842 |
| REPS2 | RALBP1 associated Eps domain containing 2 | –1.034081 | –2.047808772 | NM_004726 |
| SFRP1 | secreted frizzled-related protein 1 | –1.03507 | –2.049213072 | NM_003012 |
| AK5 | adenylate kinase 5 | –1.038029 | –2.053420367 | NM_174858 |
| PDGFRL | platelet-derived growth factor receptor-like | –1.041004333 | –2.057659596 | NM_006207 |
| PARP1 | poly (ADP-ribose) polymerase 1 | –1.041575 | –2.058473677 | NM_001618 |
| PSG4 | pregnancy specific beta-1-glycoprotein 4 | –1.055183667 | –2.077982734 | NM_002780 |
| MAGEC2 | melanoma antigen family C, 2 | –1.061285 | –2.086789385 | NM_016249 |
| TMEM71 | transmembrane protein 71 | –1.083335333 | –2.118929126 | NM_144649 |
| PCDHB16 | protocadherin beta 16 | –1.138055 | –2.20084112 | NM_020957 |
| IL6 | interleukin 6 (interferon, beta 2) | –1.148125667 | –2.216257736 | NM_000600 |
| CD24 | CD24 molecule | –1.166669333 | –2.244928246 | NM_013230 |
| SLC7A2 | solute carrier family 7 (cationic amino acid transporter, y+ system), member 2 | –1.184736 | –2.27321793 | NM_003046 |
| IL31RA | interleukin 31 receptor A | –1.188645667 | –2.279386648 | NM_139017 |
| SERPINB7 | serpin peptidase inhibitor, clade B (ovalbumin), member 7 | –1.214168333 | –2.320069998 | NM_003784 |
| EPHB2 | EPH receptor B2 | –1.240937667 | –2.363520973 | NM_017449 |
| GFPT2 | glutamine-fructose-6-phosphate transaminase 2 | –1.283321 | –2.43398622 | NM_005110 |
| MEST | mesoderm specific transcript homolog (mouse) | –1.299951333 | –2.462205767 | NM_002402 |
| TLR4 | toll-like receptor 4 | –1.361969333 | –2.570358038 | NR_024168 |
| SLC35F2 | solute carrier family 35, member F2 | –1.384453667 | –2.610730731 | NM_017515 |
| NIPAL1 | NIPA-like domain containing 1 | –1.452535333 | –2.736885981 | NM_207330 |
| EFEMP1 | EGF-containing fibulin-like extracellular matrix protein 1 | –1.490303 | –2.809479747 | NM_004105 |
| SLCO1B3 | solute carrier organic anion transporter family, member 1B3 | –1.506553667 | –2.841304923 | NM_019844 |
| ST3GAL6 | ST3 beta-galactoside alpha-2,3-sialyltransferase 6 | –1.534849 | –2.897581017 | NM_006100 |
| ANKRD1 | ankyrin repeat domain 1 (cardiac muscle) | –1.862053 | –3.635246011 | NM_014391 |
| CPA4 | carboxypeptidase A4 | –1.958202333 | –3.885774911 | NM_016352 |
| TMEM156 | transmembrane protein 156 | –1.966405333 | –3.907931917 | NM_024943 |
| LCP1 | lymphocyte cytosolic protein 1 (L-plastin) | –2.075092667 | –4.213714789 | NM_002298 |
| EPAS1 | endothelial PAS domain protein 1 | –2.364011333 | –5.14799743 | NM_001430 |
| DSP | desmoplakin | –2.397196333 | –5.267784521 | NM_004415 |
